# Biological Aging, Mortality, and Alzheimer’s Disease Related Biomarkers from Midlife to Old Age

**DOI:** 10.1093/geroni/igab046.2501

**Published:** 2021-12-17

**Authors:** Carol Franz, Erik Buchholz, Amy Yongmei Qin, Xin Tu, William Kremen

**Affiliations:** University of California San Diego, La Jolla, California, United States

## Abstract

People age at different rates and in different biological systems that may differentially contribute to accelerated decline. Better understanding of biological aging may contribute to identification of better targets for intervention. In 1005 VETSA participants we created 3 indicators of biological age: physiological age (PA), frailty, and brain age. PA included hemoglobin, glucose, lipids, height, weight, waist, systolic and diastolic blood pressure, and age. PA was calculated using the Klemera and Doubal (2006) method. The frailty index summed 37 health deficits (Jiang et al. 2017). A machine learning algorithm was used to estimate brain age across cortical and subcortical regions (Liem et al, 2017); predicted brain age subtracted from chronological age comprised the predicted brain age difference score (PBAD). Frailty and PBAD were calculated at waves 1, 2 and 3 when participants were average age 56, 62, and 68, respectively. PA markers were only available at waves 2 and 3. Outcome measures included mortality by wave 3 and scores on AD-related plasma biomarkers—Neurofilament light (NFL), Tau, and AB40 and AB42 at wave 3. Frailty at wave 1 and 2 predicted mortality. Frailty at wave 1 was significantly associated with wave 3 NFL, AB42 and AB40. Wave 2 & 3 frailty was associated with all biomarkers. Neither PA nor PBAD predicted biomarkers or mortality. The results are striking given the relatively young age of the sample. Even as early as one’s 50s, frailty in a community-dwelling sample predicted accelerated decline and mortality when the outcome age was only 66-73.

